# Association of high sensitive C-reactive protein (hsCRP) with established cardiovascular risk factors in the Indian population

**DOI:** 10.1186/1743-7075-8-19

**Published:** 2011-03-28

**Authors:** Panniyammakal Jeemon, Dorairaj Prabhakaran, Lakhmy Ramakrishnan, Ruby Gupta, F Ahmed, KR Thankappan, CC Kartha, Vivek Chaturvedi, KS Reddy

**Affiliations:** 1 Institute of Cardiovascular and Medical Sciences, University of Glasgow, Glasgow, UK; 2 Centre for Chronic Disease Control, New Delhi, India; 3 All India Institute of Medical Sciences, New Delhi, India; 4 KPC Medical College, Jadavpur, Kolkata, India; 5 Sree Chitra Tirunal Institute of Medical Sciences and Technology, Trivandrum, Kerala, India; 6 Rajiv Gandhi Center for Biotechnology, Trivandrum, India; 7 GB Pant Hospital, New Delhi, India; 8 Public Health Foundation of India, New Delhi, India

## Abstract

**Introduction:**

Inflammation, the key regulator of C-reactive protein (CRP) synthesis, plays a pivotal role in atherothrombotic cardiovascular disease.

**Methods:**

High sensitivity CRP (hsCRP) analysis was carried out in randomly selected 600 individuals from the sentinel surveillance study in Indian industrial population (SSIP). The hsCRP was measured quantitatively by turbid metric test using kits from SPINREACT, Spain. We analyzed the association between hsCRP and traditional CVD risk factors in this sub-sample.

**Results:**

Complete risk factor data and CRP levels were available from 581/600 individuals. One half (51.2%) of the study subjects were males. Mean age of the study group was 39.2 ± 11.2 years. The Pearson correlation coefficients were in the range of 0.12 for SBP (p = 0.004) to 0.55 for BMI (p < 0.001). The linear regression coefficients ranged from 0.01 for SBP, PG and TC (p < 0.001) to 0.55 for log_e_TAG (p < 0.001) after adjustment for age, sex and education. The mean of log_e_hsCRP significantly increased (P < 0.001) from individuals with ≤1 risk factors (-0.50) to individuals with three or more risk factors (0.60). In the multivariate model, the odds ratios for elevated CRP (CRP ≥ 2.6 mg/dl) were significantly elevated only in females in comparison to males (1.63, 95% CI; 1.02-2.58), overweight individuals in comparison to normal weight individuals (3.90, 95% CI; 2.34-6.44, p < 0.001), and abdominal obese individuals (1.62, 95% CI; 1.02-2.60, p = 0.04) in comparison to non-obese individuals.

**Conclusion:**

Clinical measurements of adiposity (body mass index and abdominal obesity) correlate well and can be surrogate for systemic inflammatory state of individuals.

## Introduction

While more than two third of all reported deaths are attributable to non-communicable diseases in India, close to one third of all deaths are due to cardiovascular disease (CVD) alone [1,2]. As the overall burden of CVD continues to grow, it is expected to be the leading cause of death and disability by 2020 (2). Excess risk of CVD among individuals from the Indian sub-continent, despite lower or similar levels of traditional risk factors have been documented among migrants from the Indian subcontinent, in comparison to the non-migrant host country populations [3]. Numerous novel biomarkers have been proposed to have mechanistically plausible links to clinical CVD and their risk factors, with many reported to identify people at an increased risk for future cardiovascular events independent of the presence of established risk factors [4]. However, the strength of the dose response relationship of these novel risk factors with cardiovascular events and their role in risk stratification and risk prediction need to be established more precisely [4,5].

Role of inflammation in the pathogenesis of atherosclerosis especially the associations between C-reactive protein (CRP), a plasma protein synthesized in liver, with cardiovascular risk factors and disease risk have gained much attention in the recent past [6]. CRP is a sensitive inflammatory marker and modulated by mediators of the inflammatory cascade (e.g., interleukin 6). While CRP is elevated in a variety of conditions, a link has been suggested between CRP and pathogenesis of clinical cardiovascular disease. For example, a recent meta-analysis of 54 long-term prospective studies suggests continuous association of CRP with risk of coronary heart disease (CHD), ischemic heart disease (IHD), and vascular mortality independent of conventional risk factors [7]. Anand and Yusuf recently analyzed the collective evidence of causal association between CRP and CVD and suggested CRP as a bystander (marker) of CVD rather than a causal factor [8]. However, research on association between CRP and cardiovascular risk factors and diseases may be more relevant among Indians who are at a significantly higher risk of developing insulin resistance, diabetes mellitus and CHD [9]. The relationships between CRP levels and conventional risk factors of CVD in the Indian population are described in this paper.

## Methods

### Study design and population

We carried out hsCRP analysis in 4 out of 10 industrial sites from the sentinel surveillance study in Indian industrial population (SSIP) chosen to represent the North (Delhi), South (Trivandrum), West (Nagpur) and East (Dibrugarh) regions of the country. The details of the SSIP have been described previously [10,11]. In brief, ten industrial sites across India participated in the study. All employees and their family members between the ages of 20 and 69 years were eligible to be included in the study. Risk factor data were obtained from randomly selected employees and their eligible family members (*n *= 9600 from four sites). From this group, we chose 6000 individuals by stratified random sub-sampling for blood lipids and fasting blood glucose analysis. Further, we randomly chose 600 individuals for serum hsCRP estimation from this group. The sample selection method is illustrated in Figure 1.

**Figure 1:**
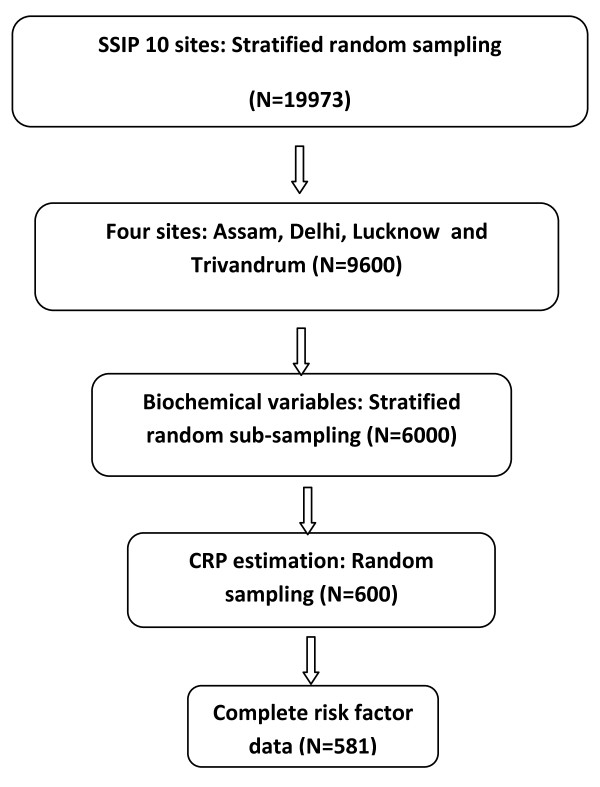
**Study flow chart**. SSIP, sentinel surveillance study in Indian industrial population

Demographic details and lifestyle habits were obtained using a structured questionnaire. The anthropometric profile was measured using standard procedures and equipments. Blood pressure was measured using automated BP monitoring equipment (Omron MX3). Two measurements were taken at least 5 minutes apart and before collecting the blood samples. Study subjects were instructed in advance not to consume any drinks and tobacco at least one hour before attending the screening clinic.

### Laboratory analysis

A minimum 8-hour fasting blood sample was collected for biochemical analysis of blood glucose, lipids and hsCRP. Biochemical assays were performed at the Department of Cardiac Biochemistry at All India Institute of Medical Sciences (AIIMS), New Delhi. Details of biochemical analysis and quality control measures are published elsewhere [10]. Briefly, glucose was analyzed by means of glucose oxidase method (GOD-PAP, Randox). Cholesterol estimation was by CHOD-PAP and triglycerides by GPO-PAP method (Randox). HDL was estimated by the precipitation method using phosphotungstate/MgCl_2_. The method entails precipitation of Apo B containing lipoproteins followed by estimation of cholesterol in the supernatant by enzymatic method.

The hsCRP was measured quantitatively by turbid metric test using kits from SPINREACT, Spain. Latex particles coated with specific anti-human CRP were agglutinated when mixed with samples containing CRP. The agglutination causes an absorbance change depending upon the CRP content in the sample. The absorbance change was quantified using calibrators of known CRP concentration (calibration curve). The linearity of the method was up to 10 mg/l. All the samples having values >10 mg/l were diluted further and reanalyzed. The intra-assay coefficient of variation was < 5% and inter-assay coefficient was <10%.

### Definitions

Current tobacco use was defined as use of any form of tobacco products (smoking and smokeless form) in the previous 30 days. Overweight was defined as body mass index (BMI) ≥ 25 kg/m^2 ^[12]. Hypertension was defined as either a systolic blood pressure (SBP) ≥ 140 mmHg, and/or a diastolic blood pressure (DBP) ≥ 90 mmHg, or on drug treatment for hypertension [13]. Diabetes was defined as either a fasting blood glucose value of ≥ 126 mg/dl [14] or on medication for diabetes. Dyslipidemia was defined as total cholesterol to high-density lipoprotein ratio (TC/HDL) of ≥4.5. Hypertriglyceridemia was defined as triglycerides (TAG) > 150 mg/dl [15]. Elevated hsCRP was defined as hsCRP levels ≥ 75^th ^percentile (CRP ≥ 2.6 mg/dl).

### Statistical analysis

Triglycerides (TAG) and hsCRP data have been transformed using the log_e _function. Pearson correlation coefficient was estimated to test the associations of CRP with other traditional risk factors of CVD (continuous variables). Linear regression coefficients were estimated for each CVD risk factors (continuous variables) using CRP as the dependent variable and after adjustment for age, sex and education. The standard deviation (SD) and 95% CI of the regression coefficients were estimated. A regression curve was fitted to demonstrate the association of CRP with each CVD risk factor. Error bars were used to illustrate the mean difference in CRP levels (bars representing CI of the mean) according to various risk factor thresholds. Logistic regression analysis was performed to calculate both bivariate and multivariate OR for elevated CRP (CRP ≥ 2.6 mg/dl, 75 percentile value of CRP in the study population) and their 95% CI. The data were analyzed by using the Statistical Package for Social Sciences Version 15 (SPSS, Inc., Chicago, IL).

## Results

### Characteristics of the study population

Complete risk factor data and CRP levels were available from 581/600 individuals. One half (51.2%) of the study subjects were males. Mean age of the study group was 39.2 years (SD = 11.2). Tobacco use was prevalent in 47.2% males and 18.6% females (Table 1). Mean BMI and waist circumference were 23.0 kg/m^2 ^(SD = 4.5) and 80.3 (SD = 12.3) cm. Systolic BP was significantly higher (p = 0.02) among males (126.6 mmHg, SD = 15.1) in comparison to females (123.6 mmHg, SD = 19.1). Mean diastolic BP was 77.2 (SD = 11.5) mmHg. Mean TC/HDL-c was significantly elevated (p = 0.04) in males (4.3, SD = 1.6) in comparison to females (4.0, SD = 1.6). Similarly, the median triglycerides levels were significantly higher in males (117.0, IQR = 79.0-164.0) in comparison to females (89.5, IQR = 66.8-126.0). While the prevalence of diabetes was 11.8% among males, it was 6.2% in females (p = 0.02). Prevalence of hypertension (32.8% Vs 29.3%, p = 0.38) and dyslipidemia (28.9% Vs 19.0%, p = 0.007) were also significantly higher in males in comparison to females. The median CRP levels were 1.1 (IQR = 0.40-2.10) and 1.2 (IQR = 0.30-3.10) in males and females, respectively.

**Table 1:** Characteristics of the study population

Variables	Males N = 305	Females N = 295	P value
Age years (mean, SD)	39.8 (11.5)	38.5 (10.8)	0.17
Current tobacco use (n, %)	144 (47.2)	54 (18.6)	<0.001
BMI Kg/m^2 ^(Mean, SD)	22.5 (3.9)	23.6 (5.1)	0.005
WC cm (Mean, SD)	82.5 (11.7)	77.9 (12.4)	<0.001
SBP mmHG (Mean, SD)	126.6 (15.1)	123.6 (19.1)	0.02
DBP mmHG (Mean, SD)	77.9 (10.9)	79.5 (12.0)	0.13
PG mg/dl (Mean, SD)	98.3 (31.4)	95.2 (32.5)	0.24
TC mg/dl (mean, SD)	174.9 (43.8)	174.2 (43.1)	0.85
TAG mg/dl (median, IQR)	117.0 (79.0-164.0)	89.5 (66.8-126.0)	<0.001
HDL-c mg/dl (mean, SD)	43.4 (11.2)	46.3 (11.7)	0.002
TC/HDL-c (mean, SD)	4.3 (1.6)	4.0 (1.6)	0.04
Diabetes (n, %)	36 (11.8)	18 (6.2)	0.02
Hypertension (n, %)	100 (32.8)	85 (29.3)	0.38
Dyslipidemia (n, %)	87 (28.9)	54 (19.0)	0.007
CRP mg/L (median, IQR)	1.1 (0.40-2.10)	1.2 (0.30-3.10)	0.30

BMI, body mass index; WC, waist circumference, SBP, systolic blood pressure; DBP, Diastolic blood pressure; PG, plasma glucose; TC, total cholesterol; TAG, triglycerides; HDL-c, high density lipoprotein cholesterol; CRP, c-reactive protein

### Association of CRP and CVD risk factors

Body mass index (BMI) in Kg/m^2^, waist circumference (WC) in cm, SBP and DBP in mmHg, plasma glucose (PG) in mg/dl, total cholesterol (TC) in mg/dl, log_e_triglycerides in mg/dl, and TC/HDL-c were positively correlated with log_e_CRP levels (table 2). The Pearson correlation coefficients (CC) were in the range of 0.12 for SBP (p = 0.004) to 0.55 for BMI (p < 0.001). The linear regression coefficients (RC) of all these variables were positive and statistically significant (table 2 and figure 2) after adjustment for age, sex and education. The RC ranged from 0.01 for SBP, PG and TC (p < 0.001) to 0.55 for log_e_TAG (p < 0.001). HDL-c was negatively correlated with logCRP (CC = -0.13, p = 0.002; RC = -0.015, P = 0.002).

**Table 2:** Association between CRP and CVD risk factors

Variables	Correlation coefficient (CC, p value)	Regression coefficient (RC, 95% CI, p value)*
BMI Kg/m^2^	0.55 (<0.001)	0.16 (0.14-0.18, <0.001)
WC cm	0.47 (<0.001)	0.05 (0.04-0.06, <0.001)
SBP mmHG	0.12 (0.004)	0.01 (0.003-0.02, 0.004)
DBP mmHG	0.18 (<0.001)	0.02 (0.01-0.03, <0.001)
PG mg/dl	0.31 (<0.001)	0.01 (0.008-0.014, <0.001)
TC mg/dl	0.32 (<0.001)	0.01 (0.008-0.12, <0.001)
log_e_TAG mg/dl	0.21 (<0.001)	0.55 (0.34-0.76, <0.001)
HDL-c mg/dl	-0.13 (0.002)	-0.015 (-0.024 to -0.005, 0.002)
TC/HDL-c	0.28 (<0.001)	0.23 (0.17-0.30, 0.001)

BMI, body mass index; WC, waist circumference; SBP, systolic blood pressure; DBP, Diastolic blood pressure; PG, plasma glucose; TC, total cholesterol; TAG, triglycerides; HDL-c, high density lipoprotein cholesterol; CRP, c-reactive protein; *Adjusted for age, sex and education.

**Figure 2:**
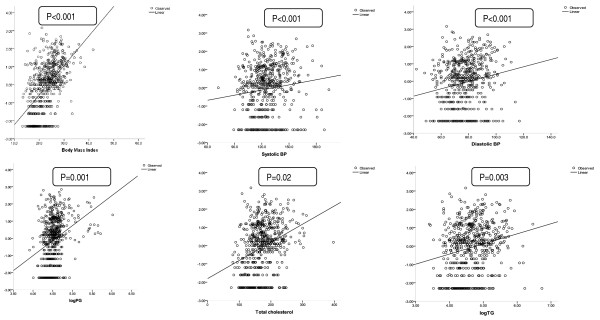
**Association between CRP concentration and conventional risk factors of CVD (regression lines)**. X axis, Log_e _of CRP; BP, blood pressure; PG, plasma glucose; TAG, triglycerides

The CRP levels were significantly lower among individual with normal blood pressure or pre-hypertension as compared to individuals with hypertension (p = 0.001), non-diabetic individuals in comparison to individuals with diabetes (p < 0.001), normal weight individuals in comparison to overweight individuals (p < 0.001), physically active in comparison to sedentary individuals (p < 0.001) and non-dyslipidemic individuals in comparison to dyslipidemic individuals (p < 0.001) after adjustment for age and sex (Figure 3). The mean of log_e_hsCRP significantly increased (P < 0.001) from individuals with ≤1 risk factors (-0.50) to individuals with three or more risk factors (0.60) (Figure 4).

**Figure 3:**
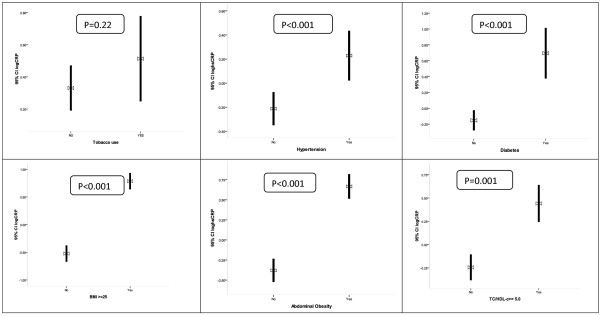
**Association between CRP and risk factors of CVD (error bars)**. BMI, body mass index; TC/HDL-c, Total cholesterol/High density lipoprotein cholesterol, hsCRP, high sensitivity C-reactive protein; hsCRP, high sensitivity C-reactive protein

**Figure 4:**
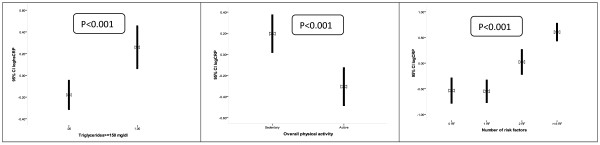
**Association between CRP and physical activity, triglycerides and clustering of risk factors of CVD (error bars)**. hsCRP, high sensitivity C-reactive protein

In the logistic regression model (table 3) the OR for elevated hsCRP were significantly higher in females in comparison to males (1.82, 95% CI; 1.24-2.67), ≥40 years in comparison to <40 years of age group (1.75, 95% CI; 1.19-2.56), overweight in comparison to normal weight subjects (6.80, 95% CI; 4.50-10.20), individuals with abdominal obesity in comparison to individuals with no abdominal obesity (3.42, 95% CI;2.31-5.07) individuals with hypertension in comparison to individuals with normal blood pressure and pre-hypertension (2.07, 95% CI; 1.40-3.05), diabetic individuals in comparison to non-diabetic individual (2.34, 95% CI; 1.31-4.18), and dyslipidemic individuals in comparison to participants with normal lipid levels (2.04, 95% CI; 1.34-3.09) in the bivariate model. In the multivariate model, the OR were significantly elevated only in females in comparison to males (1.63, 95% CI; 1.02-2.58), overweight individuals in comparison to normal weight individuals (3.90, 95% CI; 2.34-6.44, p < 0.001), and abdominal obese individuals (1.62, 95% CI; 1.02-2.60, p = 0.04) in comparison to non-obese individuals.

**Table 3:** Association between elevated CRP and CVD risk factors (logistic regression)

Variable	Elevated CRP (n, %)	Bivariate OR (95% CI, p value)	Multivariate OR (95% CI, p value)
Gender			
Male	59 (19.0)	1	1
Female	87 (29.9)	1.82 (1.24-2.66, p = 0.002)	**1.63 (1.02-2.58, p = 0.04)**
Age group			
≥40 years	89 (19.2)	1.75 (1.19-2.56, p = 0.004)	1.12 (0.72-1.76, p = 0.61)
<40 years	56 (29.3)	1	
Education*			
ES1	25 (25.0)	1	
ES2	40 (29.0)	1.22 (0.68-2.19, p = 0.45)	
ES3	53 (27.7)	1.15 (0.66-2.00, p = 0.61)	
ES4	27 (16.2)	0.58 (0.31-1.07, p = 0.08)	
Study sites*			
Lucknow	48 (29.8)	1	
Delhi	43 (27)	0.87 (0.54-1.42, p = 0.58)	
Dibrugarh	13 (9.2)	0.24 (0.12-0.64, p < 0.001)	
Trivandrum	43 (30.9)	1.05 (0.64-1.72, p = 0.83)	
Tobacco use			
No	113 (28.4)	1	1
Yes	32 (16.2)	0.50 (0.31-0.75, p = 0.001)	1.04 (0.61-1.77, p = 0.90)
Physical activity*			
Sedentary	46 (26.0)	1	
Active	49 (19.8)	0.70 (0.44-1.11, p = 0.13)	
Overweight			
BMI < 25	47 (12.0)	1	1
BMI ≥ 25	98 (48.0)	6.80 (4.5-10.2, P < 0.001)	**3.90 (2.34-6.44, p < 0.001)**
Abdominal obesity			
No	73 (17.3)	1	**1**
Yes	72 (41.6)	3.42 (2.31-5.07, P < 0.001)	**1.62 (1.02-2.60, p = 0.04)**
Hypertension			
No	82 (20.0)	1	1
Yes	63 (34.1)	2.07 (1.40-3.05, p < 0.001)	1.27 (0.80-2.02, p = 0.31)
Diabetes			
No	123 (22.7)	1	1
Yes	22 (40.7)	2.34 (1.31-4.18, p = 0.004)	1.39 (0.71-2.74, p = 0.33)
Dyslipidemia			
No	92 (20.7)	1	1
Yes	49 (34.8)	2.04 (1.34-3.09, p = 0.001)	1.45 (0.88-2.37, p = 0.15)
Hypertriglyceredimia*			
No	106 (23.5)	1	
Yes	37 (26.8)	1.19 (0.77-1.84, p = 0.43)	

*Not considered in the multilogistic regression model; CRP, c-reactive protein, OR, Odds ratio; CI, Confidence Interval; BMI, body mass index; ES1, up to primary level education, ES2, above primary level and up to secondary school education, ES3, above secondary school and up to graduation, ES4, education above graduation

## Discussion

We observed significant linear increase in hsCRP levels with body mass index, waist circumference, systolic and diastolic BP, plasma glucose, total cholesterol, TC/HDl-c and triglycerides after adjustment for age, sex and education. There was a linear increase in CRP levels with increase in mean number of risk factors (0 risk factors to ≥3 risk factors). However, in the multivariate logistic model elevated hsCRP was explained by female gender, overweight and abdominal obesity.

Statistically significant associations of hsCRP with measures of generalized and regional adiposity were previously reported in urban Indian males in the age group of 14-25 years [16]. Research data suggests that there is a strong link between overweight/adiposity with elevated CRP [17-19]. Our results are consistent with the established importance of adiposity especially the role of visceral adipose tissue (VAT) as a source of proinflammatory cytokines [20]. Obesity increases the production of proinflammatory mediators from adipose tissue T cells and contributes to insulin resistance in animal models [21]. Furthermore, low-grade proinflammatory environment and the insulin resistance associated with obesity may contribute to the down regulation of *LPIN1 *(a gene with important effects on metabolic and lipoprotein homeostasis) in adipose tissue, leading to a worse metabolic profile [22].

hsCRP levels were significantly elevated in individuals with multiple risk factors of CVD in this population in comparison to individuals with no risk factors. This association underscores the likely relationship that well-established risk factors contribute to the inflammatory process. However, a causal relationship can not be established because of the cross-sectional nature of present study. It is highly relevant to further investigate the association of CRP levels with measures of insulin resistance (overweight, fasting plasma glucose, diabetes, hypertension and clustering of risk factors) in this population. Recent evidence suggests that CRP plays a major role in the patho-physiologic processes associated with the metabolic syndrome (a cluster of CVD risk factors). For example, High levels of CRP have been shown to be an independent predictor of cardiovascular risk for all degrees of severity of the metabolic syndrome [23]. Furthermore, several studies demonstrate that CRP can be used to predict the development of type 2 diabetes mellitus [24-27]. For example, in the Women's Health Study [24], the relative risk of developing diabetes among women in the highest quartiles of CRP was significantly high (15.7;95% CI, 6.5-37.9) in comparison to women in the lowest quartiles of CRP, even after adjusting for body mass index, family history of diabetes mellitus, smoking, and other factors.

To the best of our knowledge this is the first study from the Indian population living in India showing association with CRP levels and other traditional risk factors of CVD in the age group of 20-69 years. Our data is consistent with the large meta-analysis data of 54 prospective studies worldwide (7), and data from adult migrant Indians in UK [28] and USA [29]. The increased predisposition of CHD among individuals from the Indian sub-continent, inability of traditional risk factors to fully explain the excess CHD risk among Indians, and relatively high exposure to repeated, persistent and lifelong infections in this population highlights the importance of studying CRP as a risk marker of CVD in the Indian population. Prospective studies to understand the independent relationship of CRP levels and their interaction with CVD risk factors to cardiovascular endpoints in this population are warranted.

## Conclusion

CRP levels and traditional cardiovascular risk factors are correlated with each other in the Indian population. Clinical measurements of adiposity (body mass index and abdominal obesity) readily provide data elucidating the systemic inflammatory state of individuals.

## Competing interests

PJ is supported by a Wellcome Trust Capacity Strengthening Strategic Award to the Public Health Foundation of India and a consortium of UK universities. The surveillance study was funded by Ministry of Health and Family Welfare, Government of India and World Health Organization. DP received research support/grants from Duke Clinical Research Institute, Eli Lilly & Company, Merck, Sharpe & Dohme, Unilever

## Authors' contributions

PJ wrote the statistical analysis plan, carried out the analysis and drafted the manuscript. PJ, DP, LR, VC and KSR participated in the design of the study and reviewed the manuscript. RG and LR performed the biochemical analysis, participated in the design and coordination of biochemical component of the study. KRT, FU and CCK organized data collection at different sites. PJ and VC were responsible for data management. All authors read and approved the final manuscript.
